# Traditional Chinese Medicine Injections for Diabetic Retinopathy: A Systematic Review and Network Meta-Analysis of Randomized Controlled Trials

**DOI:** 10.1089/jicm.2021.0392

**Published:** 2022-12-07

**Authors:** Wu Sun, Junnan Li, Xiaoling Yan, Liang Liao, Shimeng Li, Xueyao Wang, Caiyin Xiao, Mengqiu Shang, Guojun Chao, Jian Zhou

**Affiliations:** ^1^ Beijing University of Chinese Medicine, Beijing, China.; ^2^ Department of Ophthalmology, Dongfang Hospital, Beijing University of Chinese Medicine, Beijing, China.; ^3^ Retinal Department, Eye Hospital, Chinese Academy of Chinese Medical Sciences, Beijing, China.

**Keywords:** Traditional Chinese Medicine injection, diabetic retinopathy, network meta-analysis, systematic review

## Abstract

**Background:**

The aim of this study was to compare the efficacy of different injected Traditional Chinese Medicines in the treatment of diabetic retinopathy (DR) and to provide a reference for the selection of adjuvant therapy for DR.

**Content:**

Related literature in multiple biological databases and websites was searched up to April 15, 2022, without language and publication time restrictions. A Bayesian network meta-analysis was used to analyze the included studies.

**Summary:**

Compared with conventional treatment, the combined use of injected Traditional Chinese Medicines, including astragalus, danhong, *Ginkgo biloba* extract powder, ginkgo leaf extract and dipyridamole (GLED), ligustrazine (LIG), mailuoning, puerarin, safflower, shuxuetong, safflower yellow sodium chloride, and xueshuantong (XST), can significantly improve the clinical effectiveness in patients with DR, while LIG, XST, and GLED can improve vision. The strength of the evidence ranged from high to very low.

**Outlook:**

In patients with DR, the combination of multiple injected Traditional Chinese Medicines is more effective than conventional treatment; some of these medicines may also improve visual acuity. This study may provide a good resource and reference for the selection of adjuvant therapy for DR.

## Introduction

As a common complication of diabetes, diabetic retinopathy (DR) can severely affect patients' vision and even lead to blindness.^1^ Approximately 75% of patients with type 1 diabetes and 50% of patients with type 2 diabetes develop DR.^2^ DR is the leading cause of vision loss among working-age adults worldwide.^3,4^

The current first-line treatment of DR includes panretinal photocoagulation (PRP) and antivascular endothelial growth factor (anti-VEGF) therapy.^5^ However, PRP has serious side effects, including a reduced visual field and color vision and worsening of DR.^6^ In addition, 15% of proliferative DR (PDR) cases progress to blindness.^7^ The emergence of anti-VEGF therapy has shown significant clinical benefits in patients with DR; however, many patients failed to achieve clinically meaningful improvement in their vision.^8^

In addition, the risk of treatment-induced complications, such as infectious endophthalmitis, increased intraocular pressure, and retinal detachment, occurs more frequently as the patient receives more injections.^8,9^ Therefore, establishment of adjuvant therapies for treatment of DR is needed.

In China, Traditional Chinese Medicine injections (TCMIs) are widely used to treat DR, especially in medical communities and Chinese medicine hospitals. A number of studies found that TCMIs can increase the effective rate of treatment of DR and improve patients' vision.^10–14^ While there are varieties of TCMIs for treatment of DR, their efficacy rates have not yet been compared.

This study conducted a systematic review and network meta-analysis on the efficacy and safety of different TCMIs in the treatment of DR, aiming to compare their efficacy and provide new evidence for complementary treatment options for DR.

## Methods

This research has been registered with PROSPERO (registration no. CRD42021248555) and was written strictly in accordance with PRISMA for network meta-analyses.^15^ See Supplementary Material S1 for the details of PRISMA.

### Search strategy and study selection

The literature related to treatment of DR with TCMIs was identified by searching the following databases from inception to April 15, 2022: Embase, MEDLINE, PubMed, Cochrane Library, China National Knowledge Infrastructure, Wanfang, China Biomedical Literature Database, and Technical Journal Database. The following websites were also searched to identify potentially related literature: www.google.cn/, www.baidu.com, www.clinicaltrialsregister.eu, and www.clinicaltrials.gov

There were no restrictions on the language and publication date of the literature. The search strategy is shown in Supplementary Material S2.1.

All randomized controlled trials (RCTs) on the treatment of DR with TCMIs were included. The intervention measures for the observation group included different TCMIs, and the conventional treatment group as the main control included basic treatments such as blood sugar control and blood pressure control, as well as laser treatment and anti-VEGF treatment.

In this study, the authors define basic treatments, including blood sugar control and blood pressure control, as passive control interventions (PCIs) and basic treatments containing laser treatment or anti-VEGF treatment as active control interventions (ACIs). Other types of TCMIs were also included as controls in some of these studies. There were no restrictions on the duration and frequency of interventions and the dosage of drugs.

The main results included clinical efficacy rates and best-corrected visual acuity (BCVA). The criteria for judging effectiveness were based on the Guiding Principles for Clinical Research of New Chinese Medicines^16^ and Guidelines for Diagnosis and Treatment of Diabetic Retinopathy Combined with Disease and Syndrome^17^ and are shown in Supplementary Material S2.2. The secondary result was the side effects. Studies involving Chinese herbal medicine and studies with incorrect data and/or no results (after communicating with the authors) were excluded.

### Data extraction and quality assessment

After removing duplicates, preliminary screening was performed by reading the abstracts and titles of the remaining studies. According to the inclusion and exclusion criteria, the full text of the qualified literature was then read for further screening. The eligible literature was finally included and the following relevant data were extracted: (1) participant information, including age, sex, and number and staging of DR; (2) type of intervention and course of treatment; and (3) interesting outcome indicators.

If there was missing and/or incorrect information, the authors communicated with the original author and asked for relevant information. Two researchers independently conducted the literature search process.

According to the Cochrane risk of bias tool^18^ recommended by Review Manager, version 5.3, two investigators individually performed the risk of bias assessment based on the following items: sequence generation, allocation hiding, blinding of participants, and blinding of personnel and outcome data, incomplete data evaluation, selective reporting, and other types of biases. Each item was divided into three levels: low, high, and unclear.

The overall risk of bias in each included study was evaluated according to the table in Supplementary Material S3.1.^19^ The inconsistencies and differences in the aforementioned steps were resolved through a discussion with a third researcher.

### Data synthesis and analysis

A frequency-based paired meta-analysis was used to analyze all direct comparisons, with dichotomous data recorded using the risk ratio (RR) and its 95% confidence interval (CI) and continuous data recorded using standard mean deviation (SMD) and its 95% CI for effect size statistics. *I*^2^ values and *χ*^2^ tests were used to assess heterogeneity,^20^ and significant heterogeneity was defined when *I*^2^ values were >50% or *p* < 0.10.

The authors used the random effects model in the Bayesian framework to conduct a network meta-analysis and generated network plots for each outcome indicator. RR or SMD and its 95% credibility interval were used for effect size statistics. The authors assumed that the network met the consistency requirements and had common heterogeneity parameters in all processing comparisons.

The transitivity hypothesis was evaluated by comparing the clinical and methodological characteristics of the included studies (e.g., patient characteristics, trial design).^21^ After randomly constructing four parallel Markov chains to simulate accurate estimates of the statistical model, each chain went through 50,000 iterations.^22^ The first 20,000 iterations were discarded to ensure that deviation from the initial value was minimized by the time the chain reached its target distribution.^23^

The Brooks–Gelman–Rubin diagnosis was used to check model convergence.^24^ Inconsistencies between direct and indirect evidence were explored by the design-by-treatment^25^ and node-splitting methods.^26^ The surface under the cumulative ranking curve (SUCRA) was used to rank each treatment, with the highest SUCRA value considered more likely to be the most effective.^27^

In addition, subgroup analyses were performed according to different classifications of DR and treatment durations. Comparison-adjusted funnel plots were used to assess the effects of small sample sizes and publication bias.

These analyses were performed using the GeMTC package (version 1.0-1) and Meta package (version 4.18-2) of the RStudio program (version 4.1) and Stata software (version 14). The running code of the network meta-analysis is shown in Supplementary Material S4.

### Assessment of the quality of evidence

The grading of recommendations, assessment, development, and evaluation network meta-evidence evaluation system^28^ was used to evaluate the quality of each outcome, including these six items: within-study bias, reporting bias, indirectness, imprecision, heterogeneity, and incoherence. The strength of evidence was divided into four levels (high, medium, low, and very low).

## Results

### Search strategy and study selection

A total of 3346 articles were retrieved; 3335 were retrieved through the database and 11 were retrieved through the webpage and trial registration center. After excluding 969 duplicate articles, 2377 articles remained. After reading the titles and abstracts of the articles, 2231 articles were excluded, and the remaining 99 articles were read in full.

**FIG. 1.**
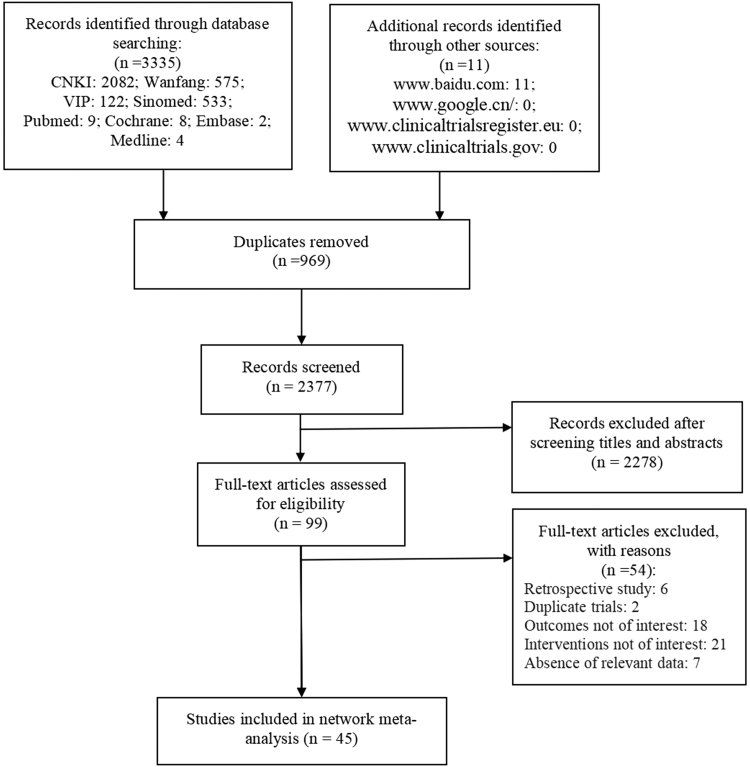
The process of selecting studies for inclusion in the meta-analysis.

After removing duplicate publications, retrospective literature, and literature containing inappropriate interventions, uninteresting outcome indicators, and/or missing key data, 45 articles were finally included.^29–73^ The specific screening process is shown in Figure 1.

### Study characteristics and quality assessments

All of the included researches were two-arm studies, including 44 articles and 1 academic article.^34^ A total of 4134 participants and 5369 eyes were involved. The studies were conducted in China between 1998 and 2021. The age range of patients was 38–78 years, and the proportion of women was 44.5%. In total, 23 studies (51.1%) were conducted on patients with non-PDR (NPDR), 7 studies (15.6%) involved patients with PDR, and the remaining studies did not mention classification information of DR.

The TCMIs included in the study involved 15 injections, including astragalus (AST), danhong (DH), danshen (DS), danshen–ligustrazine (DSL), *Ginkgo biloba* extract powder (GBEP), ginkgo leaf extract and dipyridamole (GLED), kudiezi (KDZ), ligustrazine (LIG), mailuoning (MLN), puerarin (PUE), safflower (SAF), shuxuening (SXN), shuxuetong (SXT), safflower yellow sodium chloride (SYSC), and xueshuantong/xuesaitong (XST).

All TCMIs were used on the basis of PCIs, and the conventional treatment in three studies (6.7%) involved laser therapy. Forty-two of the studies were compared with placebos, and three studies performed direct comparisons between different TCMIs.

Four of the included studies chose inappropriate randomization methods, and all of the studies did not mention concealment of random assignment or blinding of participants and outcome evaluators. None of the studies was found to have significant case shedding and reporting bias. Five studies provided information on funding, and none of the studies reported funding from pharmaceutical companies.

Overall, 4 studies (8.9%) were rated as high risk; 31 (68.9%) as moderate risk; and 10 (22.2%) as low risk. See Supplementary Material S5 for details of the included literature, and see Supplementary Material S3.2 and S3.3 for the evaluation of risk of bias.

### Results of meta-analysis

The results of the overall paired meta-analysis of clinical efficacy rates did not show significant heterogeneity (*I*^2^ = 3%, *p* = 0.42), while the resulting BCVA values were significantly heterogeneous (*I*^2^ = 89%, *p* < 0.01). By observing the funnel plot of clinical efficacy rates and BCVA, no publication bias was detected. Supplementary Material S6 shows the detailed results of the paired meta-analysis.

**FIG. 2.**
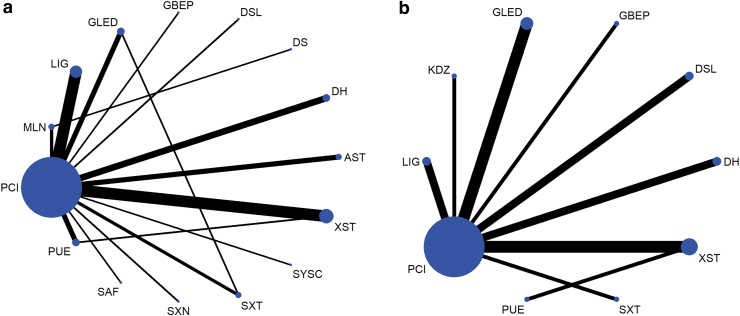
Network of evidence of all the trials for clinical efficacy rates **(a)** and BCVA **(b)**. The size of nodes represents the number of patients included in the corresponding intervention. The thickness of lines represents the number of studies directly compared between the two interventions. BCVA, best-corrected visual acuity.

**FIG. 3.**
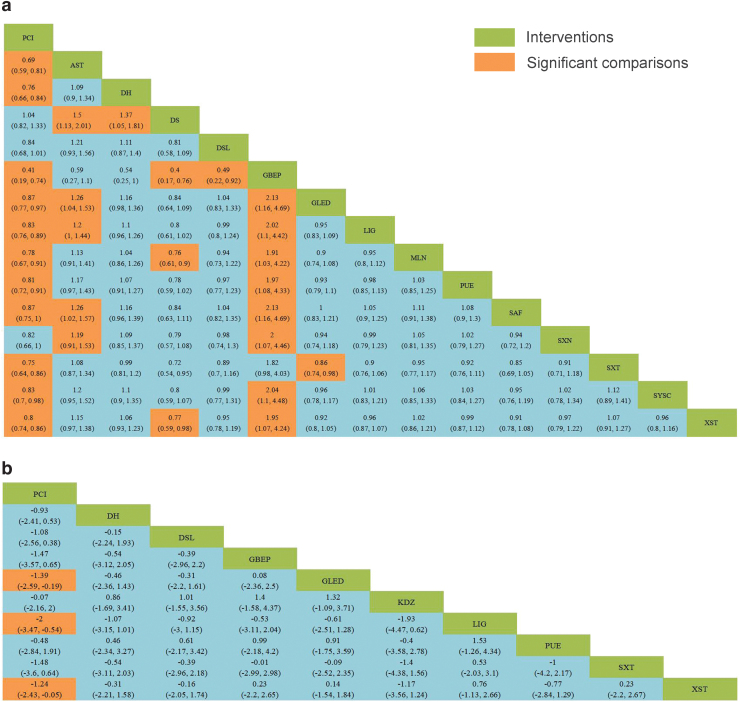
Relative effect sizes of clinical efficacy rates **(a)** and BCVA **(b)** according to network meta-analysis. BCVA, best-corrected visual acuity.

Figure 2 shows the results of the network plot. With the exception of DS, all of the included studies performed direct comparisons with PCIs. The comparisons between different injections were only performed between GLED and SXT, MLN and DS, and PUE and XST. Figure 3 shows the main network meta-analysis results.

Compared with PCIs, TCMIs, including AST, DH, GBEP, GLED, LIG, MLN, PUE, SAF, SXT, SYSC, and XST, can significantly improve the clinical efficacy rates of these medicinal injections in patients. In addition, GBEP was superior to other injections except DH, SXT, and AST. In terms of BCVA improvement, LIG, GLED, and XST performed better than PCIs, although the differences between different injections were not statistically significant.

**FIG. 4.**
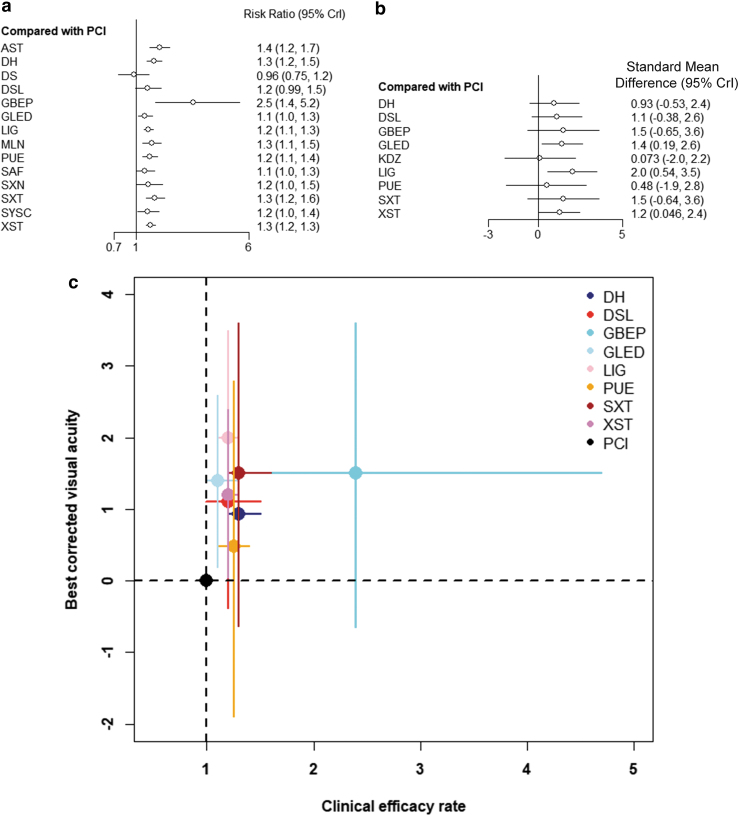
Forest plots of network meta-analysis of all TCMIs for clinical efficacy rates **(a)** and BCVA **(b)** compared with PCIs; two-dimensional graphs about clinical efficacy rates and BCVA mentioned for all TCMIs **(c)**. BCVA, best-corrected visual acuity; PCIs, passive control interventions; TCMI, Traditional Chinese Medicine injection.

As shown in Figure 4a and b, the authors also presented the relative efficacy of different TCMIs versus PCIs. In addition, they drew a two-dimensional plot of clinical effectiveness and BCVA using PCI as a reference, as shown in Figure 4c. In accordance with the ranking based on SUCRA values, GBEP was the best for improving the clinical efficacy rate, while LIG was the most effective in improving BCVA. For detailed information, see Supplementary Material S7.

Through a subgroup analysis of patients with NPDR, the authors found that compared with PCIs, SXT cannot improve the clinical effect in patients, and GLED and LIG do not have the advantage of improving BCVA. In addition, when the duration of the intervention was limited to within 30 and 30–60 days, the outcome did not change significantly. See Supplementary Material S8 for details of the subgroup analysis.

The basic treatment in three studies was ACI, and the TCMIs involved included DH, PUE, and SAF. The results of the network meta-analysis showed that none of the three drugs improved the clinical efficacy rate compared with ACIs. Supplementary Material S8.4 shows the details of the network meta-analysis.

In terms of the safety of the injection, seven studies have described adverse events and four have reported adverse reactions during treatment. One study^54^ found that their SYSC group had eight cases of pain reaction at the infusion site, which was relieved after adjusting the infusion rate. Another study^67^ encountered two cases of dizziness, and one case of nausea with vomiting occurred in the LIG group during infusion.

In addition, one study^47^ had four cases of adverse reactions in the SXT group and four in the PCI group, while in another study,^36^ there were two cases of adverse reactions in the XST group and seven in the PCI group; neither study described the specifics of the events.

## Discussion

This study found that compared with PCIs, TCMIs, including AST, DH, GBEP, GLED, LIG, MLN, PUE, SAF, SXT, SYSC, and XST, improved the clinical condition of patients with DR, while GLED, LIG, and XST improved patients' BCVA. According to SUCRA, GBEP was ranked the best in improving efficiency, while LIG was ranked the best in improving visual acuity.

The study population of the included literature involved patients with NPDR and PDR who were aged 38–78 years. The included population in all of the studies was Asian; thus, further exploration is needed to investigate other races. Part of the included population of the study used PRP therapy, but because there were a limited number of studies, the efficacy of TCMIs in this population is still uncertain. Since most studies did not report adverse events, the authors did not have sufficient information to evaluate the safety of these injections.

In the subgroup analysis of patients with NPDR, the outcome of some drugs changed. Considering the differences in the pathological changes of DR at different stages, the results regarding patients with PDR may change. Additionally, although some studies involved patients in the proliferative phase (15.6%), the authors were unable to conduct a comprehensive analysis of the use of TCMIs due to the limited number of studies included, and the ranking and application of the results need to be carefully considered in this population.

The authors also conducted a subgroup analysis based on the course of treatment and found that whether the intervention time was less than 30 days or within 30–60 days, the outcome did not change significantly. Since most interventions lack studies with intervention duration longer than 30 days, the authors were unable to compare benefits between the two courses. Therefore, it remains unclear whether there is any benefit for long-term treatment.

A few previous reports found that *G. biloba* extract can effectively improve the clinical effective rate in DR patients.^74–77^ *G. biloba* extract has been proven to be an antioxidant and free radical scavenger, platelet-activating factor inhibitor, vasodilator, membrane stabilizer, and metabolic regulator.^76,77^ *G. biloba* extract exerts therapeutic effects in DR mostly related to its vasoregulatory and antioxidant abilities, which can effectively improve retinal vascular blood flow and stabilize cell membranes, thereby reducing retinal edema, hemorrhage, and exudation.^78–80^

It has also been recommended as an adjunctive therapy in DR-related guidelines.^81^ However, this study could not establish the advantage of *G. biloba* extract in improving visual acuity, which was inconsistent with a previous study.^74^ The authors speculate that the limitations of the inclusion criteria regarding the administration route contributed to this discrepancy.

The authors found that LIG is superior in improving visual acuity. Related studies found that improvement of vision by LIG is related to the improvement of vascular endothelial function and macular edema in patients with DR.^82,83^ LIG can significantly increase the levels of total antioxidant capacity and superoxide dismutase, reduce malondialdehyde levels, and enhance the antioxidant capacity of retinal cells^68,84–87^; this effect is enhanced with an increase of the drug dose.^85^

While most studies have reported the efficacy of LIG in patients with NPDR, one study reported the effect of LIG in PDR,^67^ which can improve the vision of patients. Some RCTs reported that LIG can downregulate the expression of hypoxia inducible factor-1 and VEGF, which may play an important role in the treatment of PDR.^51,82,88^

In addition, the authors found that XST injection in the treatment of NPDR^89,90^ can increase the clinical efficacy rate and improve visual acuity. A previous study found that XST reduces the pathological changes of DR by regulating the complement and coagulation cascade and the peroxisome proliferator-activated receptors signaling pathway, thereby improving the hemodynamic and morphological changes in the retina of diabetic rats.^91^ In addition, the inhibitory effect on inflammation and apoptosis may also be one of its mechanisms.^92^

The authors also noted that there are some reports on the efficacy of XST capsules in the treatment of PDR, which can reduce edema and eliminate vitreous hemorrhage.^93,94^ The network pharmacology analysis showed that the mechanism of XST for PDR was mainly related to regulation of oxidative stress as well as regulation of blood vessels and blood coagulation.^93^

However, the authors did not find any relevant research on XST injection in the treatment of PDR. Taking into account the possible differences between different dosage forms, including drug components, absorption, and metabolism pathways, the efficacy of XST injection in the proliferative phase of DR is still uncertain.

The included studies were all designed as two-arm studies with similar age and sex ratios of patients. The study population involved both NPDR and PDR patients, and all of the patients received basic treatment, including blood pressure and glucose control. The corrected funnel plot suggested the absence of publication bias. By comparing the deviance information criterion values of the consistency and inconsistency models, the authors chose the results of the consistency model (efficiency: consistency model 128.98 vs. inconsistency model 129.83; BCVA: consistency model 31.62 vs. inconsistency model 31.63).

The heterogeneity of the outcomes of the network meta-analysis was low (efficacy rate, *I*^2^ = 2%; BCVA, *I*^2^ = 1%). In addition, the node-splitting method in Supplementary Material S3.4 could not establish the existence of local inconsistencies. As shown in Supplementary Material S9, through a comprehensive evaluation of the evidence, the level of evidence was found to range from high to very low.

### Advantages and limitations

The authors conducted a comprehensive search (as much as possible) through multiple routes, which enriched the number of included studies and types of interventions and reduced the possibility of publication bias. The authors restricted the intervention route of drugs to intravenous infusion and excluded intervention routes such as nebulization, iontophoresis, and intramuscular injection, which increased the similarity between studies.

None of the included studies have reported on funding provided by pharmaceutical companies; thus, the outcome of the included studies lacked the driving influence of commercial interests.

However, the research had the following limitations. First, many studies have not reported information about the concealment of random methods and implementation of blind methods, which reduced the interpretation of the evidence. Second, while DR is a long-term disease, there are few studies to date that have reported follow-up records of patients after intervention. The authors are uncertain about the long-term efficacy of the injection. In addition, there are few studies to date involving patients treated with PRP and no studies related to anti-VEGF therapy patients receiving TCMIs; therefore, application of TCMIs in this population needs to be confirmed by further studies.

For future trials, the authors recommend the use of quantitative outcomes (e.g., retinal thickness, BCVA) to measure efficacy, especially in patients with PDR, and encourage head-to-head studies to compare the efficacy of different TCMIs. In addition, longer follow-up periods are necessary for a more accurate assessment of the efficacy of TCMIs.

## Conclusions

Compared with conventional treatment, the combined use of injected Traditional Chinese Medicines, including AST, DH, GBEP, GLED, LIG, MLN, PUE, SAF, SXT, SYSC, and XST, can improve clinical efficiency rates in patients with DR, while LIG, XST, and GLED improve visual acuity. The strength of these findings ranged from high to very low.

In general, the authors' research shows the potential of Chinese patent medicine injection as an adjuvant treatment for DR and provides a new resource and reference for the research of new directions in treatment of DR.

## Supplementary Material

Supplemental data

Supplemental data

Supplemental data

Supplemental data

Supplemental data

Supplemental data

Supplemental data

Supplemental data

Supplemental data
