# Supplementary material for: Traditional Chinese Medicine Injections for Diabetic Retinopathy: A Systematic Review and Network Meta-Analysis of Randomized Controlled Trials
Source: J Integr Complement Med. 2022 Dec 7;28(12):927–39. doi: 10.1089/jicm.2021.0392 (PMC9805861; doi:10.1089/jicm.2021.0392)
Supplement: Supplemental data [file Suppl_MaterialS2.doc]

**Supplementary material 2: Search strategy and evaluation criteria of clinical efficacy.**

**2.1 Detail of the search strategy for PubMed.**

| NO. | Search item |
| --- | --- |
| 1# | “Diabetic Retinopathy” [MeSH] |
| 2# | “Diabetic Retinopathies [title/abstract] ” or “Retinopathies, Diabetic [title/abstract] ” or “Retinopathy, Diabetic [title/abstract]” |
| 3# | 1# or 2# |
| 4# | “Complementary therapies” [MeSH] |
| 5# | “traditional Chinese medicine [title/abstract] ” or “therapies, complementary [title/abstract] ” or “complementary medicine [title/abstract]” or “herbal therapy [title/abstract] ” or “herb therapy [title/abstract] ” or “Chinese patent medicine [title/abstract]” or “Chinese herbal drugs [title/abstract] ” or “herbal [title/abstract] ” or “Chinese patent medicine [title/abstract]” or “gegensu [title/abstract] ” or “danshen [title/abstract] ” or “chuanxiongqin [title/abstract]” or “xueshuantong [title/abstract] ” or “xuesaitong [title/abstract] ” or “danhong [title/abstract] ” or “shuxuetong [title/abstract]” or “dazhuhongjingtian [title/abstract] ” or “honghua [title/abstract] ” or “huangqi [title/abstract]” or “yinxingye [title/abstract] ” or “yinxingdamo [title/abstract] ” or “mailuoning [title/abstract]” or “guanxinning [title/abstract] ” or “dengzhanhua [title/abstract] ” or “kudiezi [title/abstract]” or “ciwujia [title/abstract] ” |
| 6# | 4# or 5# |
| 7# | “Injections” [MeSH] |
| 8# | “injection [title/abstract] ” or “injectables [title/abstract] ” or “injectable [title/abstract]” |
| 9# | 7# or 8# |
| 10# | “Randomly [title/abstract] ” or “random allocation [title/abstract] ” or “randomized [title/abstract]” or “controlled clinical trial [title/abstract] ” or “randomized controlled trial [title/abstract] ” or “placebo [title/abstract]” |
| 11# | 3# and 6# and 9#and 10# |

The search strategy will be modified as required for other electronic databases.

**2.2 Evaluation criteria of clinical efficacy.**

1. Effective:conforming to any of the following including: 1. Visual acuity improvement, 2. Fundus changes including a decrease in the number of retinal microangiomas, and a decrease in the amount of bleeding and exudation in the fundus; 3. Fundus fluorescein angiography shows the shortening of retinal circulation time, the reduction of macular edema and vascular leakage.
2. Invalid: the indicators did not meet the above standards or even deteriorated.
