# Supplementary material for: Traditional Chinese Medicine Injections for Diabetic Retinopathy: A Systematic Review and Network Meta-Analysis of Randomized Controlled Trials
Source: J Integr Complement Med. 2022 Dec 7;28(12):927–39. doi: 10.1089/jicm.2021.0392 (PMC9805861; doi:10.1089/jicm.2021.0392)

**Supplementary material 3: Risk of bias assessment of included studies and the results of node splitting method.**

**3.1 Evaluation criteria for the overall risk of bias in the included studies**

| Global rating | Items with high risk | Items with unlcear risk |
| --- | --- | --- |
| Low risk | 0 | =< 3 |
| moderate | 0 | > 3 |
| moderate | 1 | any |
| High risk | >1 | any |

**3.2 Risk of Bias Assessment of clinical efficacy rates**

**
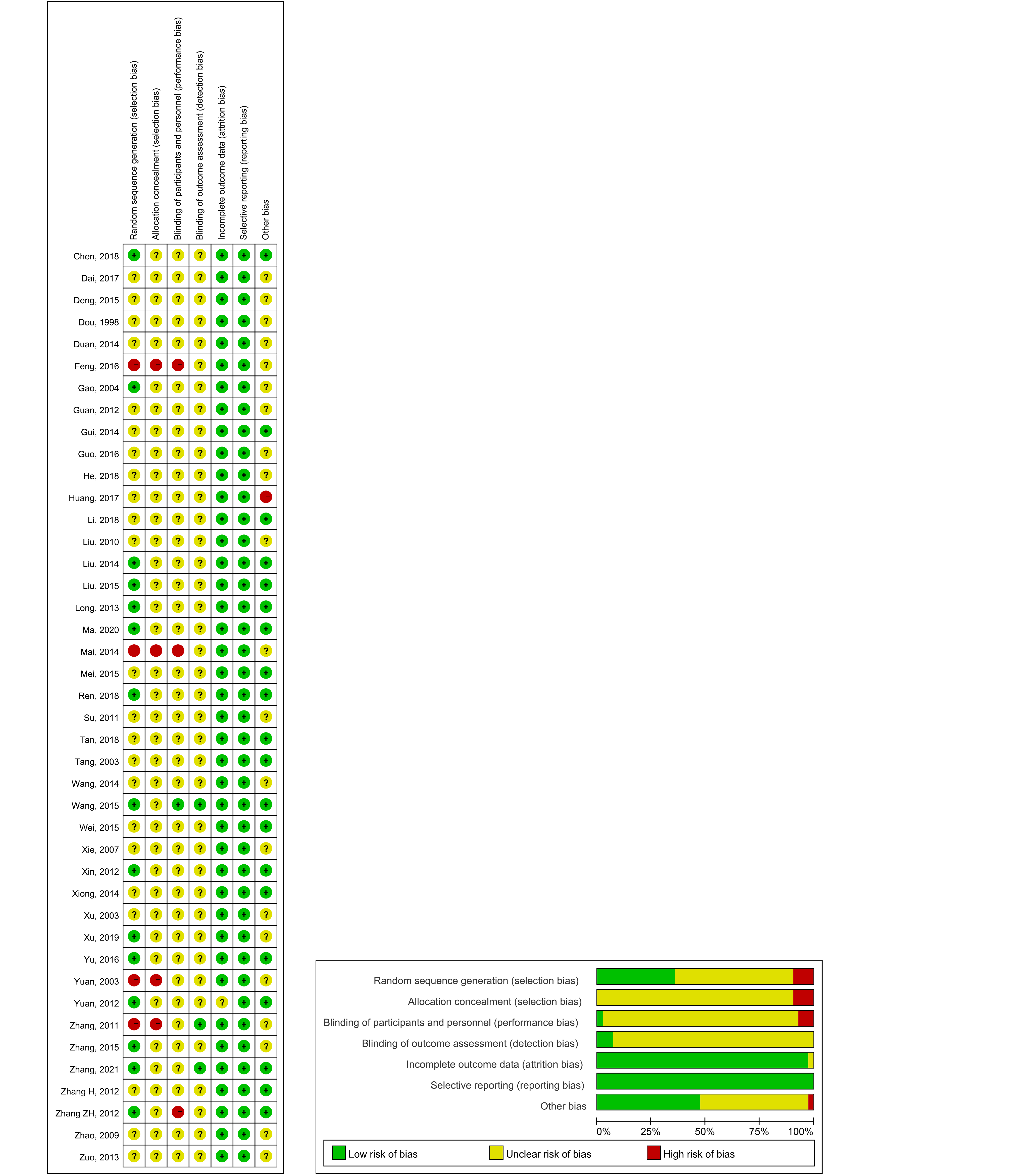
**

**3.3 Risk of Bias Assessment of best corrected visual acuity (BCVA)**

**
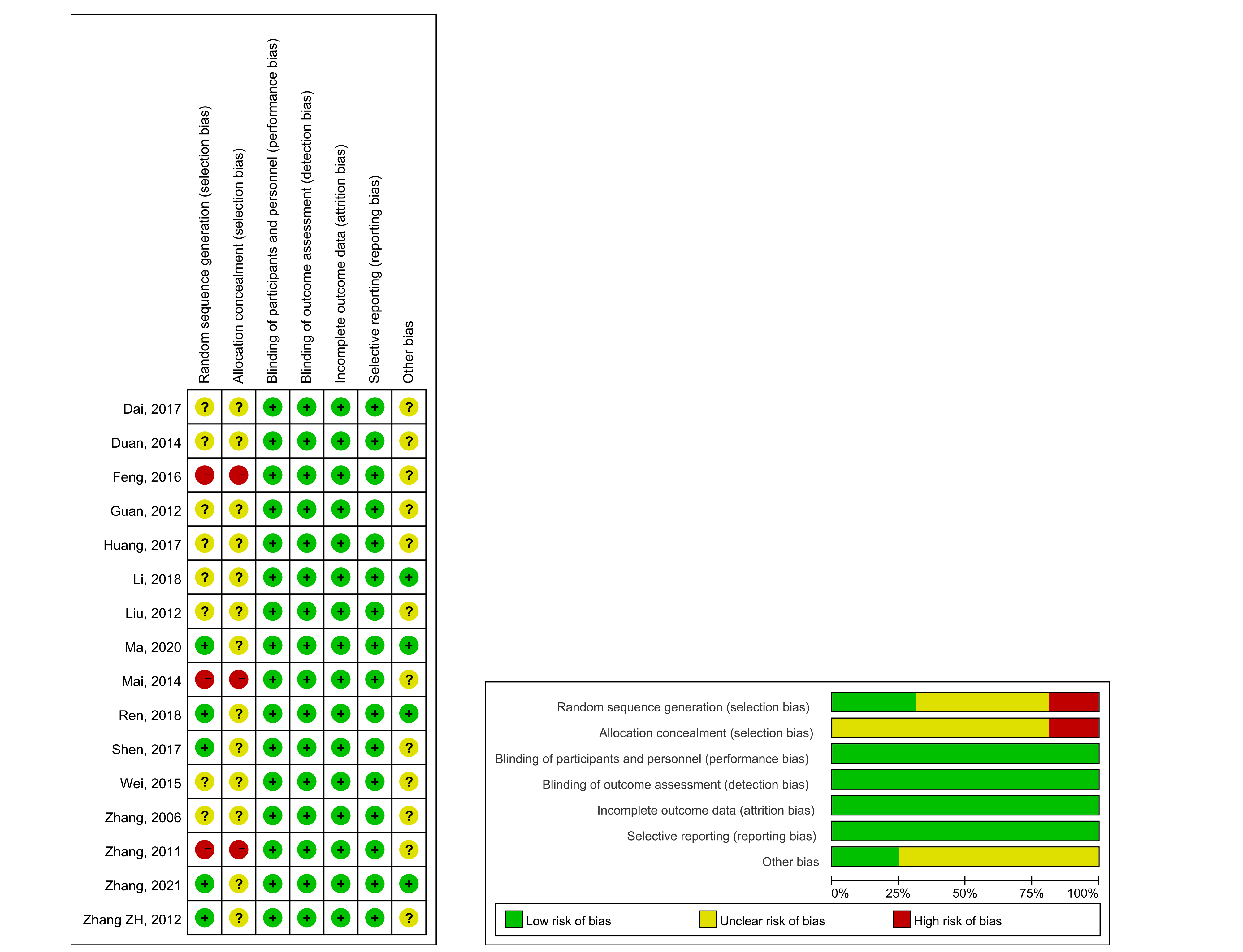
**

**3.4 Results of node splitting method for clinical efficacy rates.**


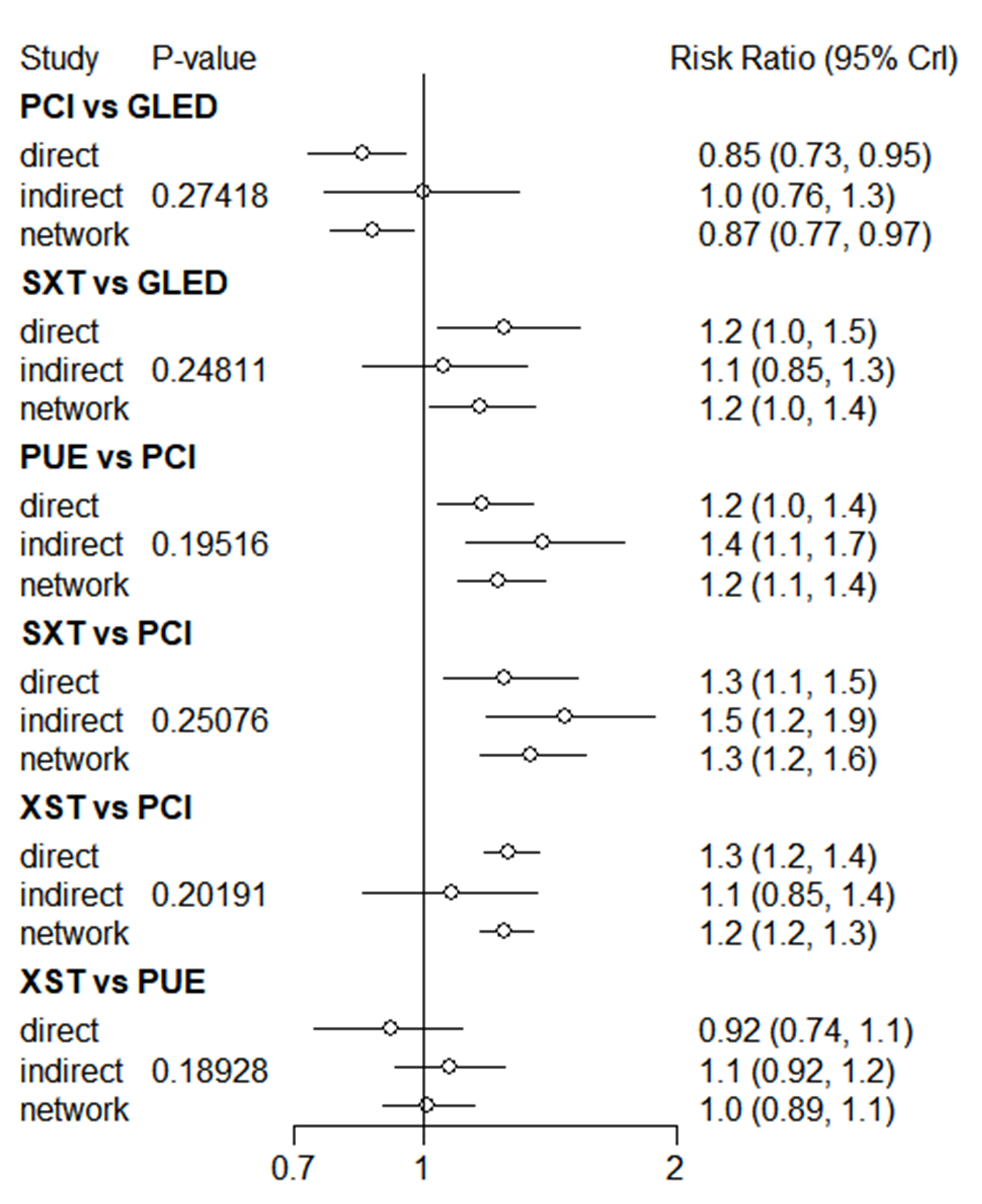

Supplement: Supplemental data [file Suppl_MaterialS3.doc]
