# Supplementary material for: Traditional Chinese Medicine Injections for Diabetic Retinopathy: A Systematic Review and Network Meta-Analysis of Randomized Controlled Trials
Source: J Integr Complement Med. 2022 Dec 7;28(12):927–39. doi: 10.1089/jicm.2021.0392 (PMC9805861; doi:10.1089/jicm.2021.0392)
Supplement: Supplemental data [file Suppl_MaterialS4.doc]

**Supplementary material 4: Running code for network meta-analysis of RStudio software.**

**# Calculation for risk ratio (RR)**

install.packages("gemtc")

library(coda)

library(gemtc)

data <- read.csv("R.csv", sep=",", header=T)

network <- mtc.network(data)

View(network)

plot(network)

model <-mtc.model(network, type="consistency", n.chain=4, likelihood="binom", link="log", linearModel="random")

results <- mtc.run(model, n.adapt = 20000, n.iter = 50000, thin = 1)

summary(results)

forest(relative.effect(results, "CT"), digits=3, xlim = c(0, 2))

# observation of the model convergence

gelman.plot(results)

# Ranking and probabilities for treatment

ranks <- rank.probability(results)

print(ranks)

# Calculation for SUCRA

sucra <- function(ranks) {

apply(ranks, 1, function(p) {

a <- length(p)

sum(cumsum(p[-a]))/(a-1)

})

}

a<-sucra(ranks)

# Nodesplit method

resultnodesplit <-mtc.nodesplit(network, n.adapt = 20000, n.iter = 50000, thin = 1, n.chain=4,likelihood="binom",link="log",linearModel="random")

# Inconsistency model

modelume <-mtc.model(network, type="ume", n.chain=4,likelihood="binom",link="log",linearModel="random")

resultsume <- mtc.run(modelume, n.adapt = 20000, n.iter = 50000, thin = 1)

summary(resultsume)

**# Calculation for standard mean deviation (SMD)**

network <- mtc.network(data.re=data, treatments=NULL, description="Network", data.ab=NULL, studies=NULL, data=NULL)

model <-mtc.model(network, type="consistency", n.chain=4, likelihood="normal", link="identity", linearModel="random")

results <- mtc.run(model, n.adapt = 20000, n.iter = 50000, thin = 1)

summary(results)

# Nodesplit method

resultnodesplit <-mtc.nodesplit(network, n.adapt = 20000, n.iter = 50000, thin = 1, n.chain=4,likelihood="normal",link="identity",linearModel="random")

# Inconsistency model

modelume <-mtc.model(network, type="ume", n.chain=4,likelihood="normal",link="identity",linearModel="random")

resultsume <- mtc.run(modelume, n.adapt = 20000, n.iter = 50000, thin = 1)

summary(resultsume)
