# Supplementary material for: Traditional Chinese Medicine Injections for Diabetic Retinopathy: A Systematic Review and Network Meta-Analysis of Randomized Controlled Trials
Source: J Integr Complement Med. 2022 Dec 7;28(12):927–39. doi: 10.1089/jicm.2021.0392 (PMC9805861; doi:10.1089/jicm.2021.0392)
Supplement: Supplemental data [file Suppl_MaterialS5.doc]

**Supplementary material 5: Characteristics of included studies.**

| Study (year) | Interventions and sample size (eyes) | Age  (years, mean ± SD) | Gender  (male/female) | DR classification | Duration (days) | Outcomes | Risk-of-bias rating |
| --- | --- | --- | --- | --- | --- | --- | --- |
| Zhang, 2021 [29] | C: PCI = 83 (83 eyes)  E: LIG = 83 (83 eyes) | C: 60.03 ± 5.91  E: 64.91 ± 6.24 | C: 48/35  E: 43/40 | NPDR | 30 | ①② | Low |
| Ma, 2020 [30] | C: PCI = 46 (46 eyes)  E: DSL = 46 (46 eyes) | C: 58.14 ± 5.92  E: 58.37 ± 5.80 | C: 27/19  E: 29/17 | PDR + NPDR | 14 | ①②③ | Low |
| Ren, 2018 [31] | C: PCI = 46 (46 eyes)  E: LIG = 46 (46 eyes) | C: 60.13 ± 4.59  E: 59.89 ± 4.67 | C: 24/22  E: 26/20 | NPDR | 30 | ①② | Low |
| Li, 2018 [32] | C: PCI = 64 (64 eyes)  E: GLED = 64 (64 eyes) | C: 66.7 ± 9.2  E: 65.1 ± 6.2 | C: 42/22  E: 43/21 | PDR + NPDR | 28 | ①② | Moderate |
| Mai, 2014 [33] | C: PCI = 20(20 eyes)  E: SXT = 36(36 eyes) | C: 64.7 ± 10.2  E: 66.1 ± 10.8 | C: 11/9  E: 21/15 | NPDR | 20 | ①② | Hihg^[a]^ |
| Zhang, 2011 [34] | C: PCI = 27(54 eyes)  E: PUE = 27(53 eyes) | C: 59.3 ± 8.8  E: 57.9 ± 8.6 | C: 16/11  E: 13/14 | NR | 40 | ①② | Hihg^[a]^ |
| Zhang ZH, 2012 [35] | C: PCI = 28 (43 eyes)  E: GLED = 54 (43 eyes) | C: 55.5  E: 55.3 | C: 15/13  E: 16/12 | NPDR | 28 | ①② | Moderate |
| Huang, 2017 [36] | C: PCI = 44 (44 eyes)  E: XST = 44 (44 eyes) | C: 56.9 ± 5.3  E: 58.2 ± 4.8 | C: 24/20  E: 25/19 | NR | 28 | ①②③ | Moderate |
| Dai, 2017 [37] | C: PCI = 40 (40 eyes)  E: DH = 40 (40 eyes) | 52.24 ± 4.75 | 45/35 | NPDR | 72 | ①② | Moderate |
| Wei, 2015 [38] | C: PCI = 32 (32 eyes)  E: DH = 32 (32 eyes) | 52.2 ± 4.8 | 38/26 | NPDR | 42 | ①② | Moderate |
| Duan, 2014 [39] | C: PCI = 72 (72 eyes)  E: XST = 72 (72 eyes) | C: 56.71 ± 11.23  E: 55.23 ± 10.87 | C: 42/30  E: 45/27 | NR | 28 | ①② | Moderate |
| Guan, 2012 [40] | C: PCI = 13 (26 eyes)  E: GBEP = 13 (26 eyes) | NR | NR | NPDR | 45 | ①② | Moderate |
| Feng, 2016 [41] | C: PCI = 32 (32 eyes)  E: GLED = 32 (32 eyes) | C: 56.8 ± 2.4  E: 56.8 ± 2.4 | C: 20/12  E: 20/12 | NPDR | 30 | ①② | Hihg^[a]^ |
| Liu, 2012 [42] | C: PCI = 32 (32 eyes)  E: DSL = 37 (37 eyes) | 38-66 | C: 17/15  E: 19/18 | NPDR | 7 | ② | Low |
| Shen, 2017 [43] | C: PCI = 43 (43 eyes)  E: XST = 43 (43 eyes) | C: 58.7 ± 3.4  E: 58.4 ± 3.1 | C: 24/19  E: 25/18 | NR | 21 | ② | Low |
| Zhang, 2006 [44] | C: PCI = 28 (28 eyes)  E: KDZ = 28 (28 eyes) | 45-73 | 22/34 | NPDR | 60 | ② | Low |
| Chen, 2018 [45] | C: PCI = 40 (40 eyes)  E: PUE = 40 (40 eyes) | C: 50.83 ± 7.84  E: 51.38 ± 8.14 | C: 21/19  E: 22/18 | NR | 28 | ①③ | Low |
| Yu, 2016 [46] | C: PCI = 40 (60 eyes)  E: DH = 41 (65 eyes) | C: 57.6 ± 11.5  E: 58.4 ± 11.7 | C: 19/21  E: 22/19 | NPDR | 56 | ① | Low |
| Guo, 2016 [47] | C: PCI = 40 (40 eyes)  E: SXT = 41 (41 eyes) | C: 57.1 ± 2.5  E: 57.8 ± 2.5 | C: 24/16  E: 25/16 | NR | 30 | ①③ | Moderate |
| Gui, 2014 [48] | C: PCI = 31(58 eyes)  E: AST = 32(60 eyes) | 63 ± 1.25 | 36/27 | NPDR | 28 | ① | Moderate |
| Deng, 2015 [49] | C: PCI = 35 (35 eyes)  E: LIG = 35 (35 eyes) | C: 56.4 ± 9.6  E: 54.9 ± 8.8 | C: 19/16  E: 21/14 | NPDR | 30 | ① | Moderate |
| Mei, 2015 [50] | C: PCI = 40 (40 eyes)  E: LIG = 40 (40 eyes) | C: 58.3 ± 2.4  E: 58.5 ± 2.6 | C: 23/17  E: 24/16 | NPDR | 30 | ① | Moderate |
| Wang, 2015 [51] | C: PCI = 30 (60 eyes)  E: LIG = 30 (60 eyes) | C: 53.71 ± 9.25  E: 51.86 ± 8.19 | C: 13/17  E: 12/18 | NPDR | 14 | ① | Low |
| Zhang, 2015 [52] | C: PCI = 40 (40 eyes)  E: XST = 40 (40 eyes) | C: 58.43 ± 8.47  E: 59.32 ± 9.12 | C: 25/15  E: 27/13 | NR | 30 | ① | Moderate |
| Wang, 2014 [53] | C: PCI = 25 (47 eyes)  E: DH = 25 (49 eyes) | C: 40.9 ± 6.6  E: 40.7 ± 6.8 | C: 13/12  E: 12/13 | NR | 42 | ① | Moderate |
| Xiong, 2014 [54] | C: PCI = 76 (76 eyes)  E: SYSC = 92 (92 eyes) | C: 59.12 ± 5.87  E: 60.45 ± 6.1 | C: 43/33  E: 55/37 | NPDR | 14 | ①③ | Moderate |
| Liu, 2014 [55] | C: PCI = 100 (170 eyes)  E: XST = 100 (150 eyes) | C: 57.11 ± 7.62  E: 56.92 ± 6.12 | C: 60/40  E: 60/40 | NPDR | 14 | ① | Low |
| Zuo, 2013 [56] | C: PCI = 48 (48 eyes)  E: SXN = 48 (48 eyes) | C: 63.2 ± 1.4  E: 63.4 ± 1.3 | C: 28/20  E: 30/18 | NPDR | 30 | ①③ | Moderate |
| Long, 2013 [57] | C: PCI = 119 (179 eyes)  E: XST = 119 (187 eyes) | 58.14 ± 7.82 | 122/116 | NPDR | 14 | ① | Low |
| Xin, 2012 [58] | C: PCI = 30 (48 eyes)  E: PUE = 30 (48 eyes) | C: 62.74 ± 10.58  E: 62.56 ± 10.24 | C: 18/12  E: 19/11 | PDR + NPDR | 20 | ① | Low |
| Yuan, 2012 [59] | C: PCI = 64 (96 eyes)  E: XST = 64 (101 eyes) | 46-69 | 66/62 | NPDR | 15 | ① | Moderate |
| Su, 2011 [60] | C: PCI = 49 (98 eyes)  E: MLN = 49 (98 eyes) | C: 59.1 ± 10.7  E: 56.3 ± 9.6 | C: 26/24  E: 24/25 | PDR + NPDR | 14 | ① | Moderate |
| Liu, 2010 [61] | C: GLED = 50 (62 eyes)  E: SXT = 50 (68 eyes) | 25-80 | 53/47 | NR | 15 | ① | Moderate |
| Xie, 2007 [62] | C: PCI = 30 (53 eyes)  E: MLN = 30 (56 eyes) | C: 58.2 ± 10.3  E: 56.7 ± 10.5 | C: 16/14  E: 17/13 | NR | 30 | ① | Moderate |
| Gao, 2004 [63] | C: PCI = 80 (156 eyes)  E: SAF = 84 (164 eyes) | C: 59 ± 9  E: 57 ± 8 | C: 39/41  E: 42/43 | NR | 40 | ① | Moderate |
| Xu, 2003 [64] | C: ACI = 21 (33 eyes)  E: APUE = 16 (21 eyes) | 27-80 | 17/20 | PDR | 14 | ① | Moderate |
| Tang, 2003 [65] | C: PCI = 30 (56 eyes)  E: AST = 30 (57 eyes) | C: 58.83  E: 60.67 | C: 15/15  E: 17/13 | NPDR | 30 | ① | Moderate |
| Yuan, 2003 [66] | C: DS = 38 (76 eyes)  E: MLN = 74 (148 eyes) | C: 57.2  E: 56.1 | C: 20/18  E: 40/34 | PDR + NPDR | 56 | ① | Hihg^[a]^ |
| Dou, 1998 [67] | C: PCI = 35 (67 eyes)  E: LIG = 52 (99 eyes) | C: 53.4 ± 10.3  E: 54.1 ± 9.8 | 39/48 | PDR + NPDR | 42 | ①③ | Moderate |
| Hao, 2018 [68] | C: PCI = 52 (98 eyes)  E: LIG = 52 (97 eyes) | C: 54.3 ± 4.3  E: 54 ± 4.4 | C: 31/21  E: 30/22 | NPDR | 14 | ① | Moderate |
| Tan, 2018 [69] | C: ACI = 41 (60 eyes)  E: ADH = 41 (63 eyes) | C: 65.02 ± 2.61  E: 65.34 ± 6.67 | C: 21/20  E: 23/18 | NPDR | 42 | ① | Moderate |
| Liu, 2015 [70] | C: ACI = 30 (44 eyes)  E: ASAF = 30 (43 eyes) | 64.73 ± 18.12 | 29/31 | NR | 15 | ① | Low |
| Zhao, 2009 [71] | C: PCI = 15 (15 eyes)  E: PUE = 15 (15 eyes) | C: 57.8 ± 9.46  E: 58.7 ± 7.61 | C: 6/9  E: 7/8 | NR | 14 | ① | Moderate |
| Zhang H, 2012 [72] | C: PCI = 30 (30 eyes)  E: AST = 30 (30 eyes) | NR | NR | NPDR | 14 | ① | Moderate |
| Xu, 2019 [73] | C: PCI = 19 (19 eyes)  E: XST = 19 (19 eyes) | C: 57 ± 2.4  E: 57.5 ± 2.6 | C: 10/9  E: 11/8 | NR | 30 | ① | Moderate |

ACI, active control interventions; ADH, ACI + danhong injection; APUE, ACI + puerarin injection; ASAF, ACI + safflower injection; AST, astragalus injection; C, control group; E, experimental group; DH, danhong injection; DR, diabetic retinopathy; DS, danshen injection; DSL, danshen-ligustrazine injection; GBEP, ginkgo biloba extract powder; GLED, ginkgo leaf extract and dipyridamole injection; KDZ, kudiezi injection; LIG, ligustrazine injection; MLN, mailuoning injection; NR, not reported; NPDR, non-proliferative diabetic retinopathy; PCI, passive control interventions; PDR, proliferative diabetic retinopathy; PUE, puerarin injection; SAF, safflower injection; SXN, shuxuening injection; SXT, shuxuetong injection; SYSC, safflower yellow sodium chloride injection; XST, xueshuantong injection; ① Clinical effective rate; ② BCVA, the best corrected visual acuity; ③ Adverse events; [a], Inappropriate method for random sequence generation.
