# Supplementary material for: Traditional Chinese Medicine Injections for Diabetic Retinopathy: A Systematic Review and Network Meta-Analysis of Randomized Controlled Trials
Source: J Integr Complement Med. 2022 Dec 7;28(12):927–39. doi: 10.1089/jicm.2021.0392 (PMC9805861; doi:10.1089/jicm.2021.0392)

**Supplementary material 6: Paired meta-analysis and comparison-adjusted funnel plot.**

**6.1 Paired meta-analysis of clinical efficacy rates**

**
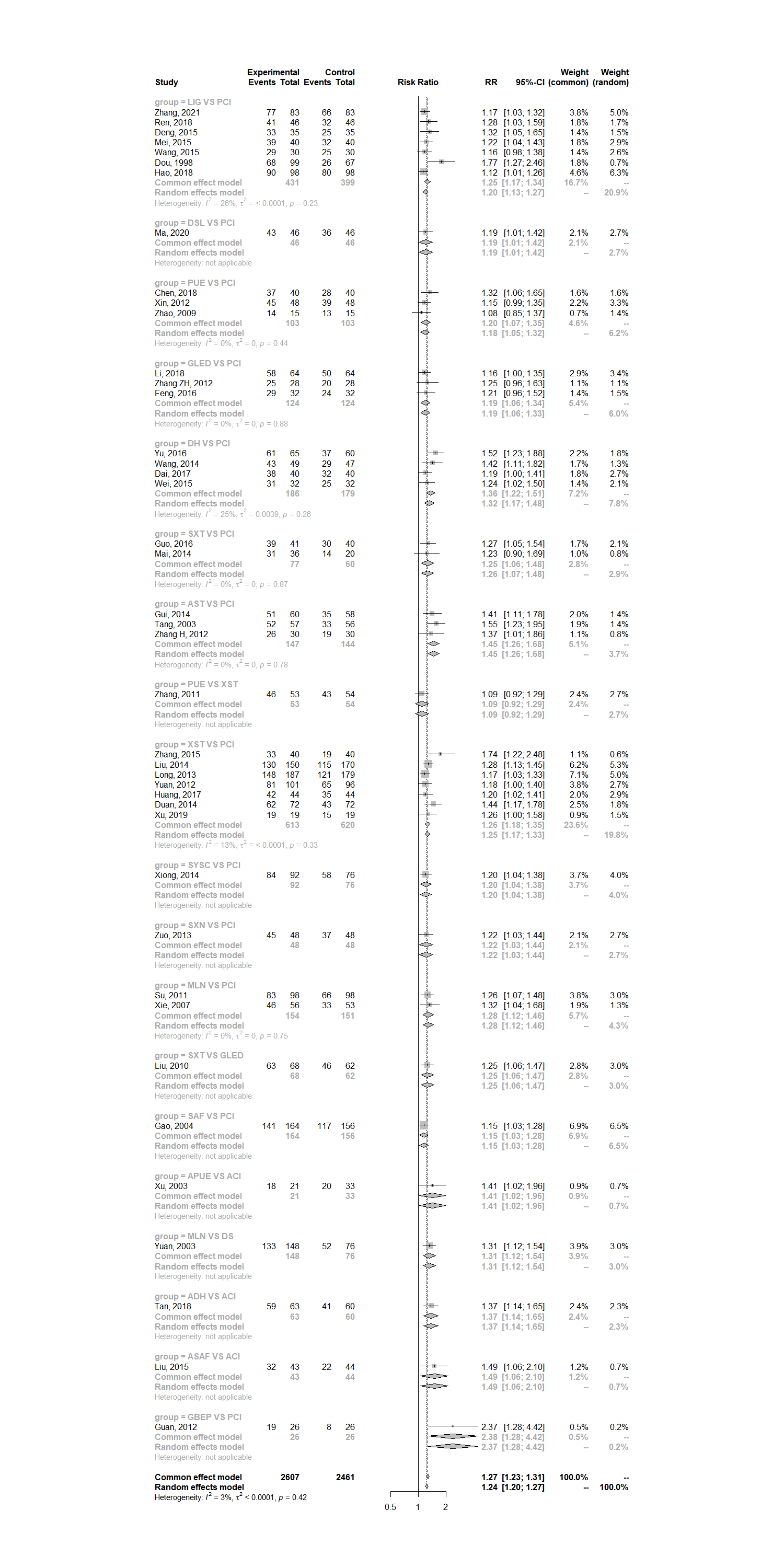
**

**6.2 Paired meta-analysis of BCVA**

**
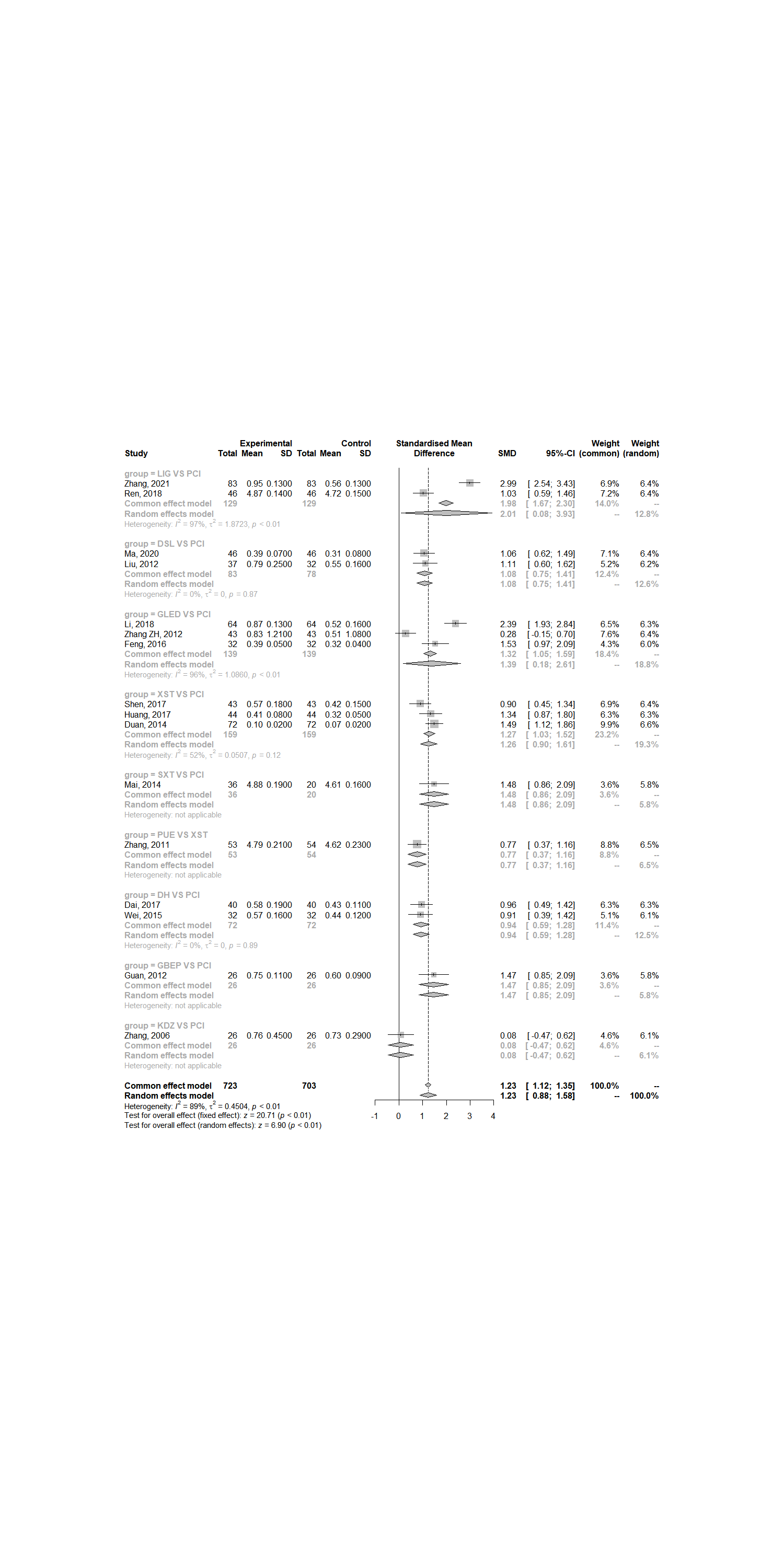
**

**6.3 The comparison-adjusted funnel plots for clinical efficacy rates (a) and BCVA (b).**


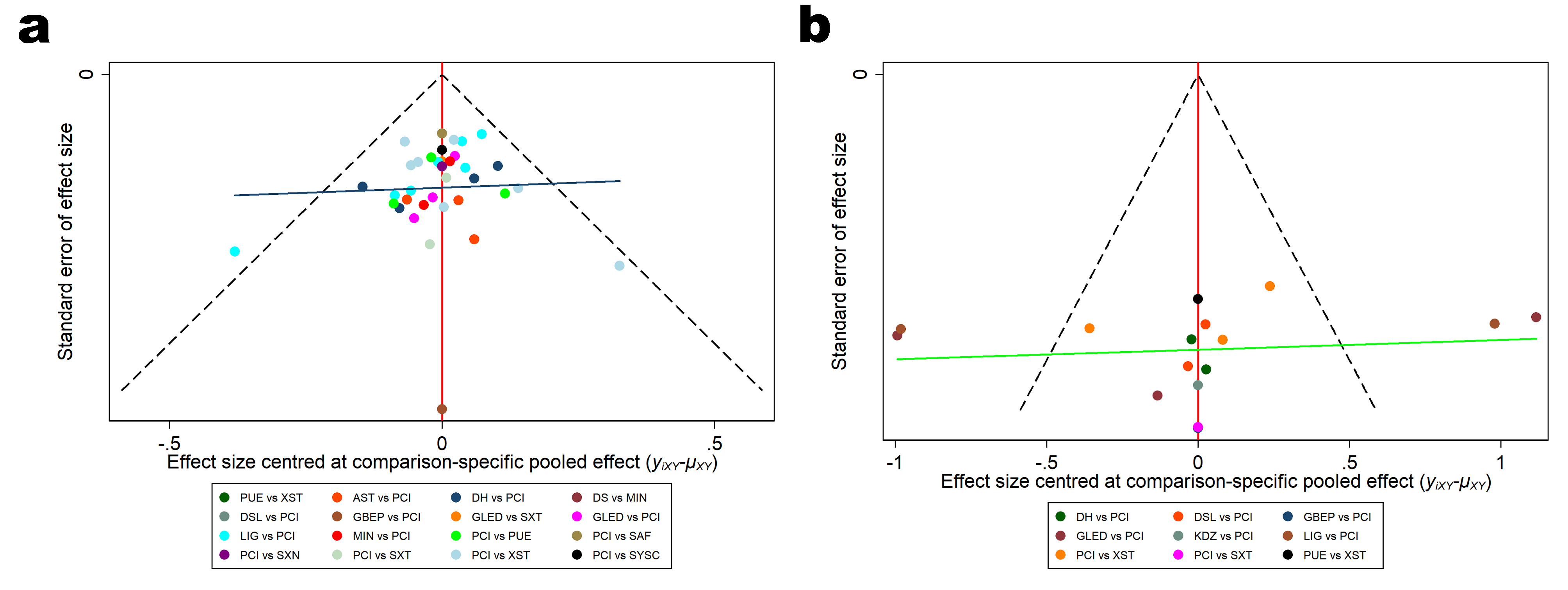

Supplement: Supplemental data [file Suppl_MaterialS6.doc]
