# Supplementary material for: Traditional Chinese Medicine Injections for Diabetic Retinopathy: A Systematic Review and Network Meta-Analysis of Randomized Controlled Trials
Source: J Integr Complement Med. 2022 Dec 7;28(12):927–39. doi: 10.1089/jicm.2021.0392 (PMC9805861; doi:10.1089/jicm.2021.0392)
Supplement: Supplemental data [file Suppl_MaterialS7.doc]

**Supplementary material 7: Probability ranking and SUCRA value ranking.**

**7.1 clinical efficacy rates**

| **Treatments** | **Rankings** | | | | | | | | | | | | | | | **SUCRA** |
| --- | --- | --- | --- | --- | --- | --- | --- | --- | --- | --- | --- | --- | --- | --- | --- | --- |
|  | 1 | 2 | 3 | 4 | 5 | 6 | 7 | 8 | 9 | 10 | 11 | 12 | 13 | 14 | 15 |  |
| GBEP | 0.94 | 0.02 | 0.01 | 0.01 | 0 | 0 | 0 | 0 | 0 | 0 | 0 | 0 | 0 | 0 | 0 | 0.90 |
| AST | 0.03 | 0.54 | 0.2 | 0.1 | 0.05 | 0.03 | 0.02 | 0.01 | 0.01 | 0 | 0 | 0 | 0 | 0 | 0 | 0.86 |
| SXT | 0.01 | 0.15 | 0.23 | 0.18 | 0.13 | 0.09 | 0.06 | 0.05 | 0.04 | 0.03 | 0.02 | 0.01 | 0 | 0 | 0 | 0.74 |
| DH | 0 | 0.09 | 0.2 | 0.21 | 0.17 | 0.12 | 0.08 | 0.05 | 0.04 | 0.02 | 0.01 | 0 | 0 | 0 | 0 | 0.72 |
| MLN | 0 | 0.07 | 0.11 | 0.14 | 0.13 | 0.11 | 0.09 | 0.08 | 0.08 | 0.06 | 0.05 | 0.04 | 0.02 | 0 | 0 | 0.61 |
| XST | 0 | 0 | 0.02 | 0.06 | 0.13 | 0.18 | 0.2 | 0.17 | 0.12 | 0.07 | 0.04 | 0.01 | 0 | 0 | 0 | 0.56 |
| PUE | 0 | 0.01 | 0.04 | 0.07 | 0.1 | 0.12 | 0.13 | 0.12 | 0.12 | 0.11 | 0.08 | 0.06 | 0.04 | 0 | 0 | 0.52 |
| SXN | 0 | 0.05 | 0.06 | 0.07 | 0.08 | 0.08 | 0.08 | 0.08 | 0.08 | 0.09 | 0.09 | 0.1 | 0.1 | 0.03 | 0.01 | 0.47 |
| LIG | 0 | 0 | 0 | 0.02 | 0.04 | 0.08 | 0.12 | 0.16 | 0.19 | 0.18 | 0.13 | 0.07 | 0.02 | 0 | 0 | 0.44 |
| SYSC | 0 | 0.02 | 0.05 | 0.06 | 0.07 | 0.07 | 0.07 | 0.08 | 0.09 | 0.11 | 0.11 | 0.12 | 0.11 | 0.03 | 0.01 | 0.434 |
| DSL | 0 | 0.04 | 0.05 | 0.06 | 0.07 | 0.06 | 0.06 | 0.07 | 0.08 | 0.09 | 0.1 | 0.12 | 0.13 | 0.05 | 0.02 | 0.428 |
| SAF | 0 | 0 | 0.01 | 0.02 | 0.02 | 0.03 | 0.05 | 0.06 | 0.08 | 0.11 | 0.15 | 0.19 | 0.21 | 0.06 | 0.01 | 0.305 |
| GLED | 0 | 0 | 0 | 0 | 0.01 | 0.02 | 0.04 | 0.06 | 0.08 | 0.12 | 0.18 | 0.23 | 0.21 | 0.04 | 0 | 0.299 |
| DS | 0 | 0 | 0 | 0 | 0 | 0 | 0.01 | 0.01 | 0.01 | 0.01 | 0.02 | 0.04 | 0.09 | 0.2 | 0.61 | 0.07 |
| PCI | 0 | 0 | 0 | 0 | 0 | 0 | 0 | 0 | 0 | 0 | 0 | 0 | 0.06 | 0.59 | 0.34 | 0.05 |

**7.2 BCVA**

| **Treatments** | **Rankings** | | | | | | | | | | **SUCRA** |
| --- | --- | --- | --- | --- | --- | --- | --- | --- | --- | --- | --- |
|  | 1 | 2 | 3 | 4 | 5 | 6 | 7 | 8 | 9 | 10 |  |
| LIG | 0.39 | 0.24 | 0.14 | 0.09 | 0.06 | 0.04 | 0.02 | 0.01 | 0 | 0 | 0.83 |
| SXT | 0.19 | 0.16 | 0.12 | 0.11 | 0.1 | 0.09 | 0.09 | 0.07 | 0.04 | 0.03 | 0.645 |
| GBEP | 0.19 | 0.16 | 0.13 | 0.11 | 0.1 | 0.09 | 0.09 | 0.07 | 0.04 | 0.03 | 0.644 |
| GLED | 0.07 | 0.14 | 0.18 | 0.18 | 0.16 | 0.12 | 0.08 | 0.04 | 0.01 | 0 | 0.64 |
| XST | 0.03 | 0.09 | 0.15 | 0.18 | 0.19 | 0.17 | 0.11 | 0.05 | 0.01 | 0 | 0.59 |
| DSL | 0.04 | 0.08 | 0.11 | 0.13 | 0.15 | 0.16 | 0.15 | 0.1 | 0.05 | 0.02 | 0.52 |
| DH | 0.03 | 0.06 | 0.09 | 0.11 | 0.13 | 0.16 | 0.18 | 0.14 | 0.07 | 0.04 | 0.46 |
| PUE | 0.04 | 0.05 | 0.05 | 0.06 | 0.07 | 0.09 | 0.12 | 0.16 | 0.14 | 0.22 | 0.33 |
| KDZ | 0.01 | 0.02 | 0.03 | 0.03 | 0.05 | 0.06 | 0.09 | 0.14 | 0.19 | 0.37 | 0.22 |
| PCI | 0 | 0 | 0 | 0 | 0 | 0.01 | 0.06 | 0.22 | 0.43 | 0.27 | 0.12 |
