# Supplementary material for: Traditional Chinese Medicine Injections for Diabetic Retinopathy: A Systematic Review and Network Meta-Analysis of Randomized Controlled Trials
Source: J Integr Complement Med. 2022 Dec 7;28(12):927–39. doi: 10.1089/jicm.2021.0392 (PMC9805861; doi:10.1089/jicm.2021.0392)
Supplement: Supplemental data [file Suppl_MaterialS8.doc]

**Supplementary material 8: Subgroup analysis**

**8.1 Subgroup analysis for non-proliferative diabetic retinopathy (NPDR)**

**
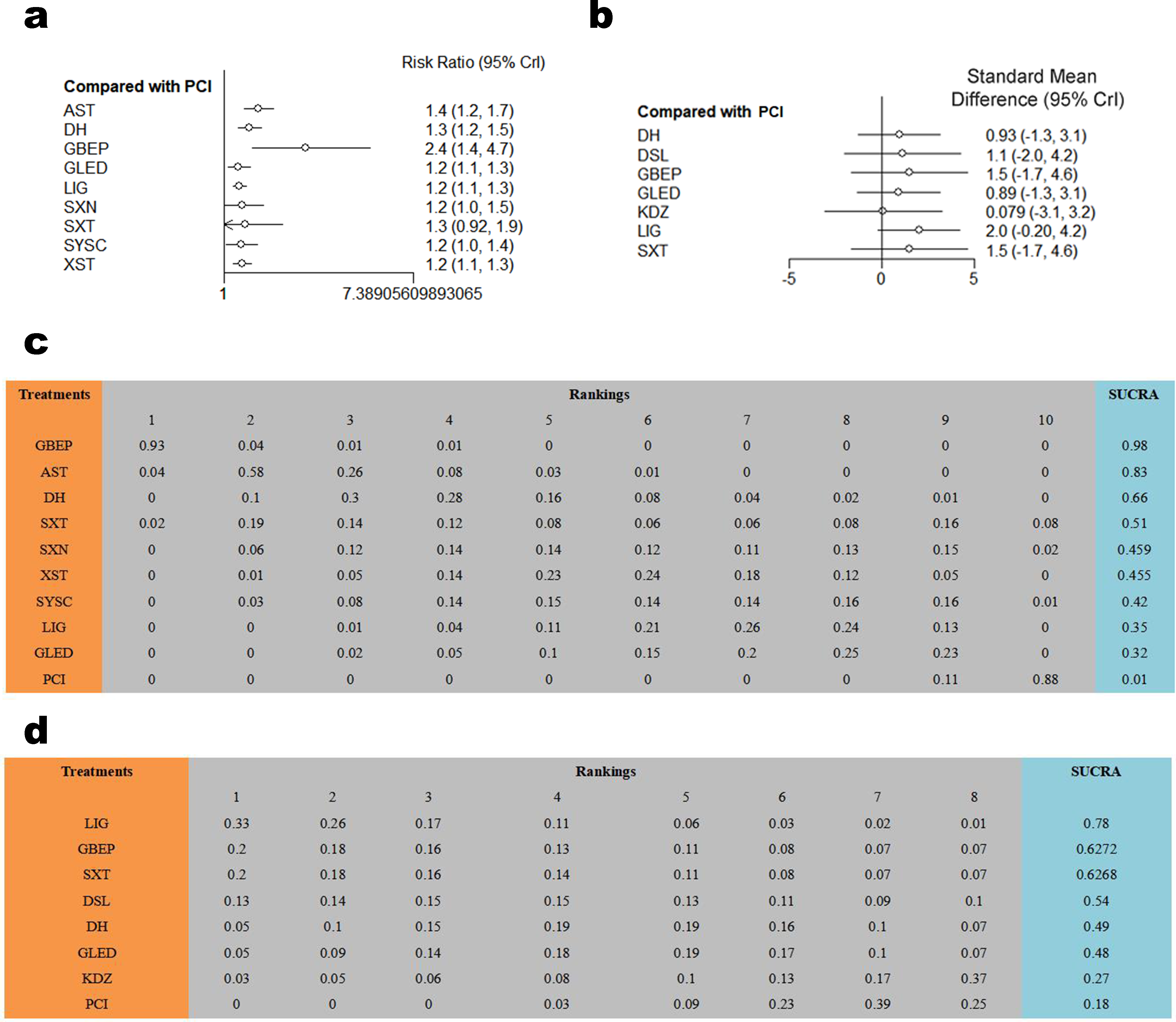
**

Forest plot for clinical efficacy rates (a) and BCVA (b); Probability ranking and SUCRA value ranking for clinical efficacy rates (c) and BCVA (d).

**8.2 Subgroup analysis based on the intervention course (≤ 30 days)**

**
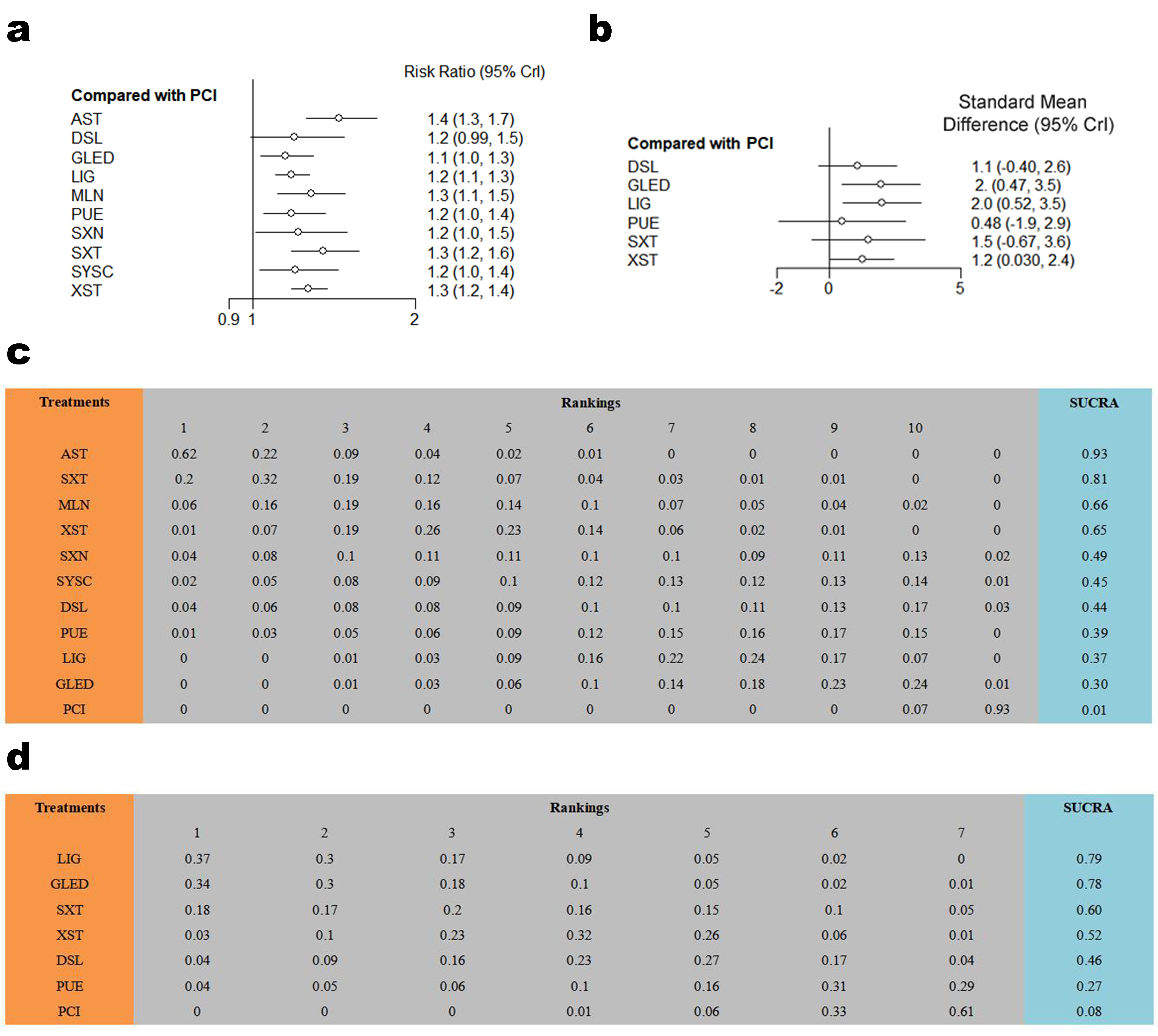
**

Forest plot for clinical efficacy rates (a) and BCVA (b); Probability ranking and SUCRA value ranking for clinical efficacy rates (c) and BCVA (d).

**8.3 Subgroup analysis based on the intervention course (30-60 days)**

**
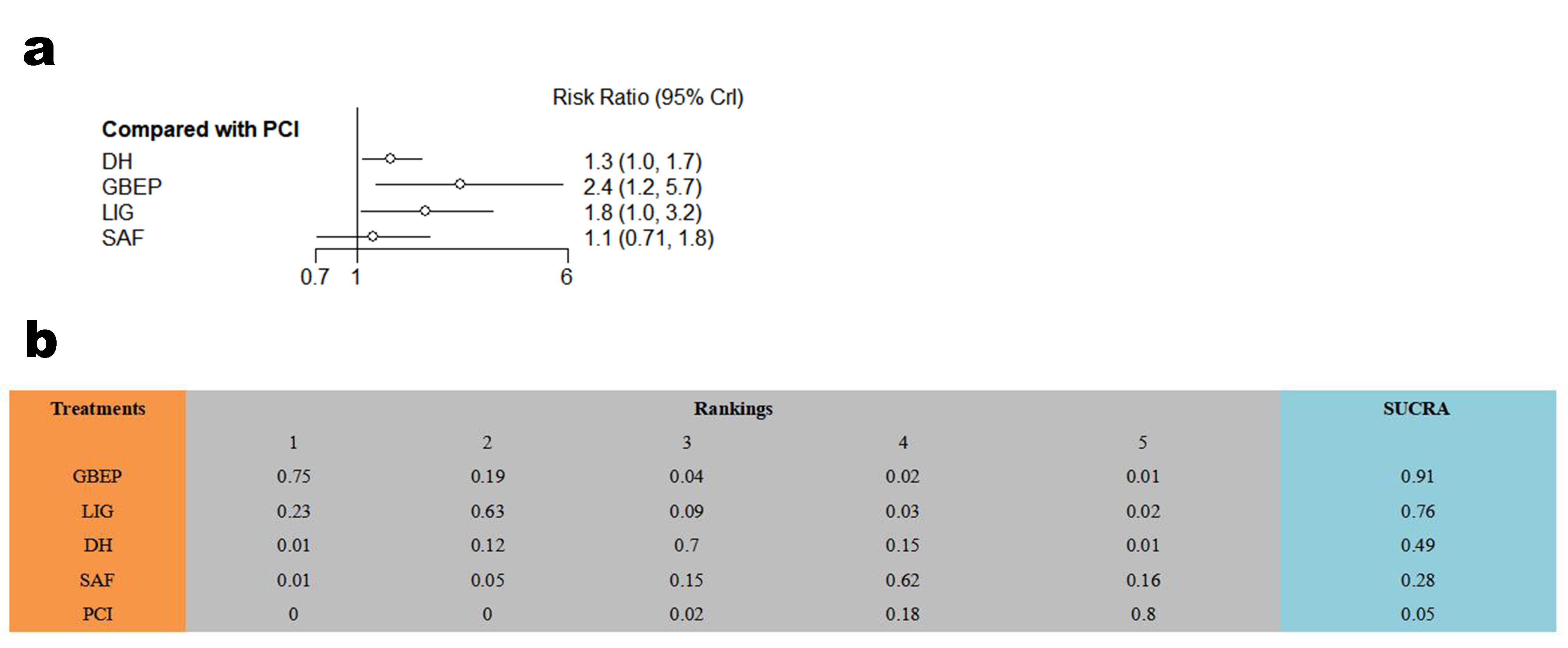
**

Forest plot for clinical efficacy rates (a); Probability ranking and SUCRA value ranking for clinical efficacy rates (b).

**8.4 The network meta-analysis results of studies involving active control interventions (ACI)**

**
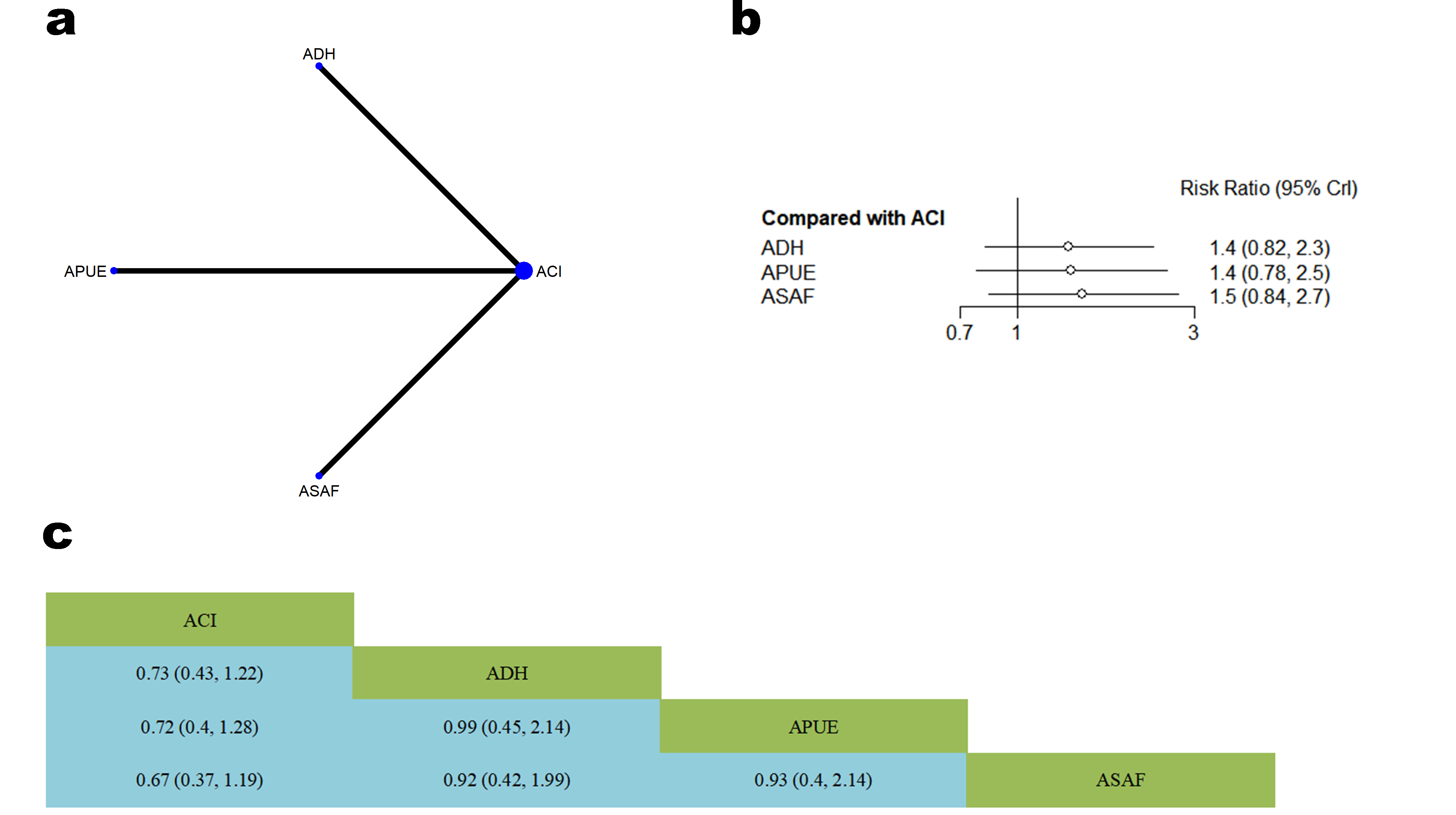
**

Network of evidence of the trials involving ACI for clinical efficacy rates (a); Forest plot for clinical efficacy rates (b); Relative effect sizes of clinical efficacy rates (c); ACI, active control interventions; ADH, ACI + danhong injection; APUE, ACI + puerarin injection; ASAF, ACI + safflower injection.
