# Supplementary material for: Traditional Chinese Medicine Injections for Diabetic Retinopathy: A Systematic Review and Network Meta-Analysis of Randomized Controlled Trials
Source: J Integr Complement Med. 2022 Dec 7;28(12):927–39. doi: 10.1089/jicm.2021.0392 (PMC9805861; doi:10.1089/jicm.2021.0392)
Supplement: Supplemental data [file Suppl_MaterialS9.doc]

**Supplement 9: Evidence evaluation of network meta-analysis**

**9.1 Clnical efficacy rates**

| Comparison | Number of studies | Within-study bias | Reporting bias | Indirectness | Imprecision | Heterogeneity | Incoherence | Confidence rating |
| --- | --- | --- | --- | --- | --- | --- | --- | --- |
| AST:PCI | 3 | Some concerns | Low risk | No concerns | No concerns | No concerns | No concerns | Moderate |
| PCI:DH | 4 | Some concerns | Low risk | No concerns | No concerns | No concerns | No concerns | Moderate |
| PCI:DSL | 1 | No concerns | Low risk | No concerns | No concerns | Major concerns | No concerns | Low |
| PCI:GBEP | 1 | Some concerns | Low risk | No concerns | No concerns | No concerns | No concerns | Moderate |
| PCI:GLED | 3 | Some concerns | Low risk | No concerns | No concerns | No concerns | No concerns | Moderate |
| PCI:LIG | 7 | Some concerns | Low risk | No concerns | No concerns | No concerns | No concerns | Moderate |
| PCI:MLN | 2 | Some concerns | Low risk | No concerns | No concerns | No concerns | No concerns | Moderate |
| PCI:PUE | 3 | Some concerns | Low risk | No concerns | No concerns | No concerns | No concerns | Moderate |
| PCI:SAF | 1 | Some concerns | Low risk | No concerns | No concerns | No concerns | No concerns | Moderate |
| PCI:SXN | 1 | Some concerns | Low risk | No concerns | No concerns | No concerns | No concerns | Moderate |
| PCI:SXT | 2 | Some concerns | Low risk | No concerns | No concerns | No concerns | No concerns | Moderate |
| PCI:SYSC | 1 | Some concerns | Low risk | No concerns | No concerns | No concerns | No concerns | Moderate |
| PCI:XST | 7 | No concerns | Low risk | No concerns | No concerns | No concerns | No concerns | High |
| PCI:DS | 0 | Major concerns | Low risk | No concerns | Major concerns | No concerns | No concerns | Very low |

**9.2 BCVA**

| Comparison | Number of studies | Within-study bias | Reporting bias | Indirectness | Imprecision | Heterogeneity | Incoherence | Confidence rating |
| --- | --- | --- | --- | --- | --- | --- | --- | --- |
| PCI:DH | 2 | No concerns | Low risk | No concerns | Major concerns | No concerns | Major concerns | Low |
| PCI:DSL | 2 | No concerns | Low risk | No concerns | Major concerns | No concerns | Major concerns | Low |
| PCI:GBEP | 1 | No concerns | Low risk | No concerns | Major concerns | No concerns | Major concerns | Low |
| PCI:GLED | 3 | Some concerns | Low risk | No concerns | No concerns | Major concerns | Major concerns | Low |
| PCI:KDZ | 1 | No concerns | Low risk | No concerns | Major concerns | No concerns | Major concerns | Low |
| PCI:LIG | 2 | No concerns | Low risk | No concerns | No concerns | Major concerns | Major concerns | Low |
| PCI:SXT | 1 | Major concerns | Low risk | No concerns | Major concerns | No concerns | Major concerns | Very low |
| PCI:XST | 3 | No concerns | Low risk | No concerns | No concerns | Major concerns | Major concerns | Low |
| PCI:PUE | 0 | Some concerns | Low risk | No concerns | No concerns | Major concerns | Major concerns | Very low |
|  |  |  |  |  |  |  |  |  |
